# Supplementary figures and images for: The Integrity of the HMR complex is necessary for centromeric binding and reproductive isolation in Drosophila
Source: PLoS Genet. 2021 Aug 23;17(8):e1009744. doi: 10.1371/journal.pgen.1009744 (PMC8412352; doi:10.1371/journal.pgen.1009744)

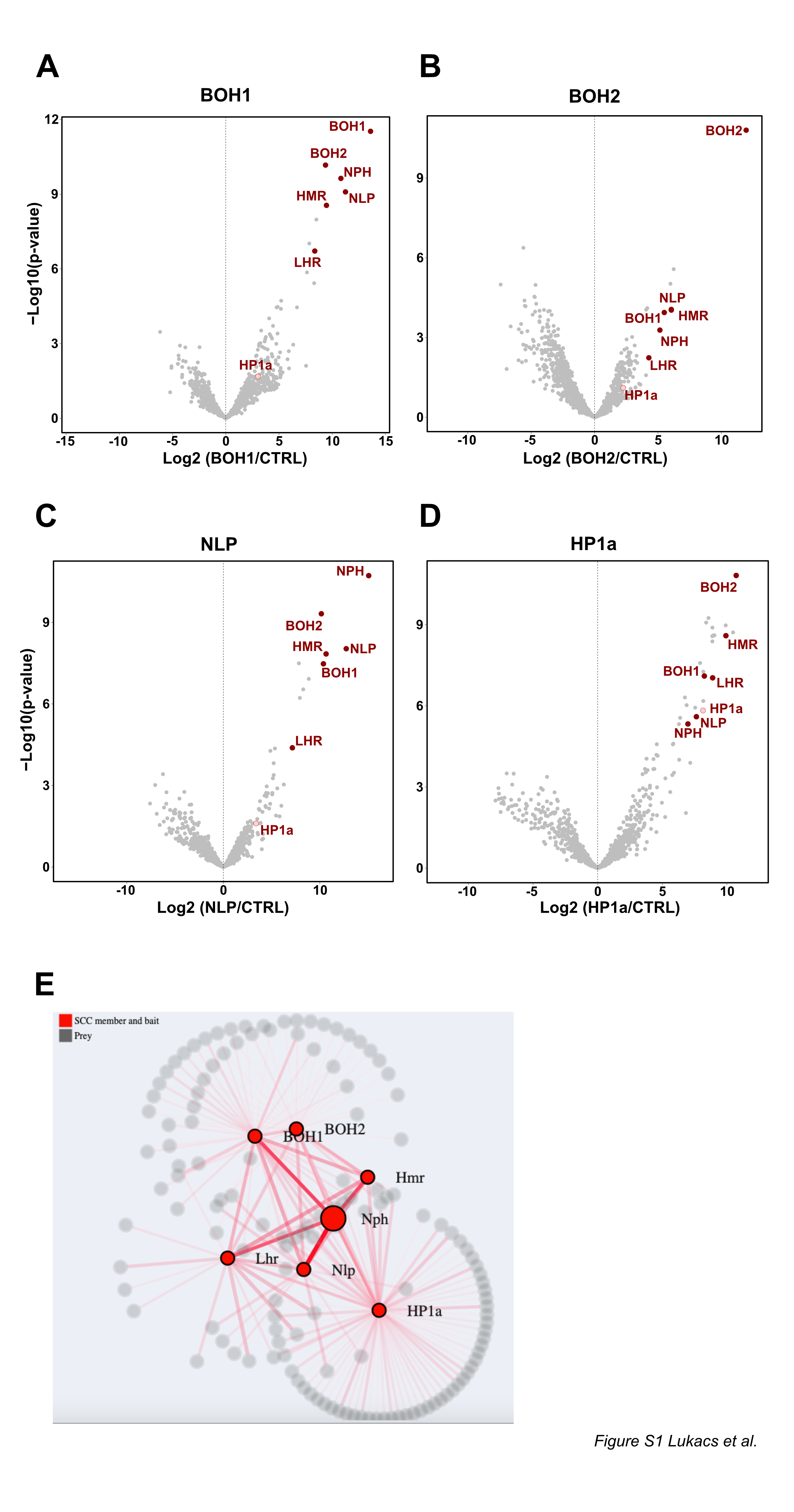

Supplement: S1 Fig — (A)—(D) HMR complex components are consistently enriched in reciprocal IPs. Volcano plots showing the interactome resulting from the AP-MS of the HMR complex components BOH1 (n = 4), BOH2 (n = 5), NLP (n = 3) and HP1a (n = 4) against a “mock” purification. The HMR complex subunits are labeled in red. X-axis: log2 fold-change of factor enrichment in IPs against mock purification (CTRL). Y-axis: significance of enrichment given as–log10 p-value calculated with a linear model. A list of the unlabeled additional bait-specific interactors is provided in S2 Table. (E) Network plot showing a highly connected HMR complex surrounded by subunit-specific interactors. Enriched proteins from each AP-MS experiment from HMR complex components were first selected (cut off: log2FC > 2.5, p-adjusted < 0.05) and integrated in an interaction network drawn with force directed layout in D3.js and R. Nodes represent proteins significantly interacting with at least one of the HMR complex components. Edges represent physical connections experimentally detected in this work. HMR complex subunits (and baits) are labelled in red. Interactive volcano plots and interaction network are available at the following (URL). (TIFF) [file pgen.1009744.s001.tiff]

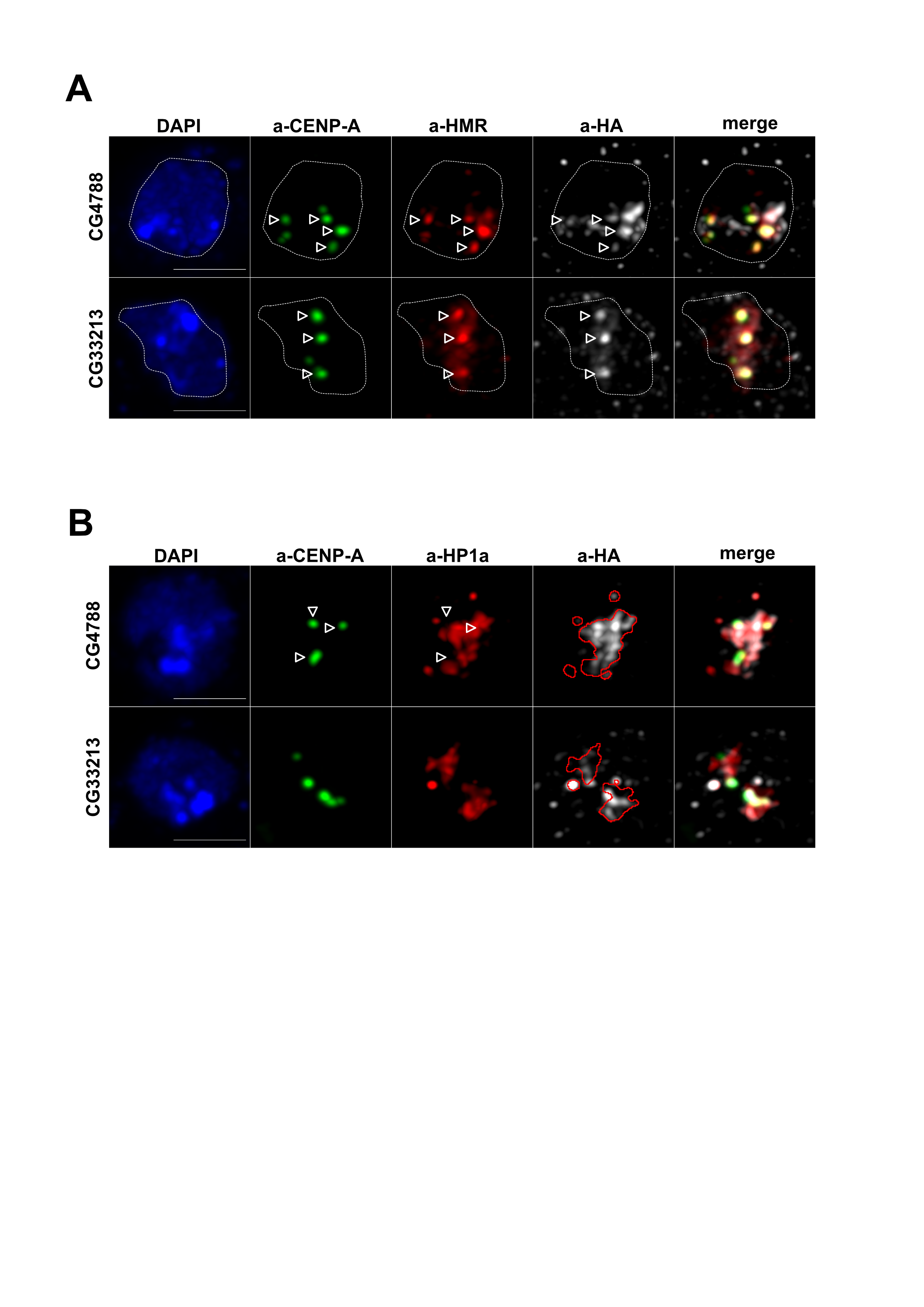

Supplement: S2 Fig — (A) BOH1 and BOH2 colocalize with HMR and CID in SL2 cells. Immunofluorescence images of cells expressing FLAG-HA-BOH2 (upper panel) or FLAG-HA-BOH1 (lower panel) showing the co-staining of HA-BOH2 or HA-BOH1, respectively, with CENP-A (centromeres) and HMR. (B) BOH1 and BOH2 colocalize with HP1a and CID in SL2 cells. Immunofluorescence images of cells expressing FLAG-HA-BOH2 (upper panel) or FLAG-HA-BOH1 (lower panel) showing the co-staining of HA-BOH2 or HA-BOH1, respectively, with CID (centromeres) and HP1a (pericentromeric chromatin). For (A) and (B) size bar indicates 3 μm, DAPI staining indicates nuclei. (TIFF) [file pgen.1009744.s002.tiff]

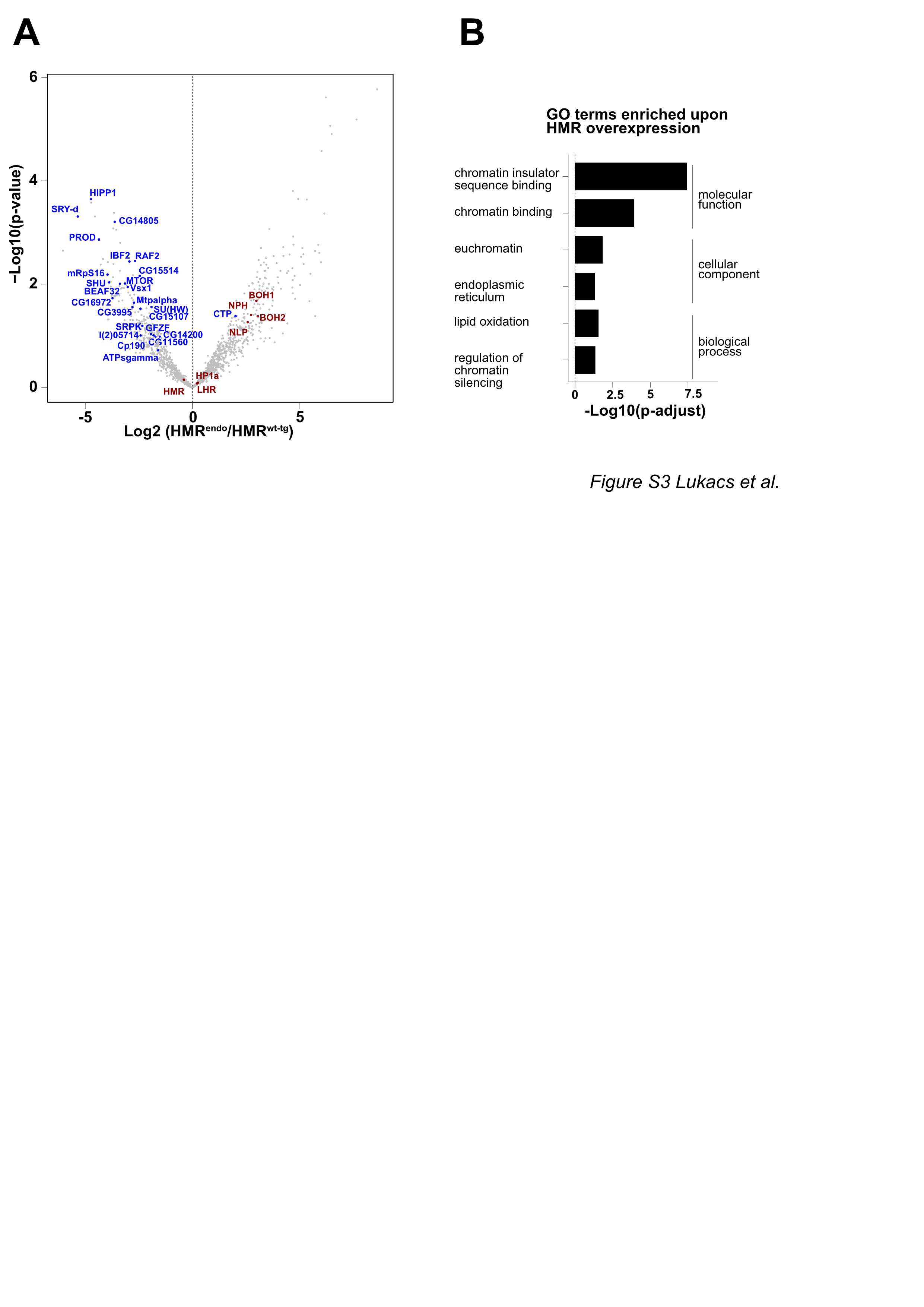

Supplement: S3 Fig — (A) Volcano plot highlighting novel interactions gained by HMR upon overexpression. X-axis: log2 fold-change of FLAG-HMRendo IPs (right side of the plot) vs FLAG-HMR+ IPs (left side of the plot). Y-axis: significance of enrichment given as–log10 p-value calculated with a linear model. HMR core complex subunits are labelled in red, novel factors enriched upon HMR overexpression are labelled in blue. Unlabeled additional bait-specific interactors are listed in S3 Table. (B) GO terms enriched upon overexpression of HMR. In (A) and (B) proteins were labelled or considered for GO search only if enriched in HMR+ or HMRendo vs CTRL (p < 0.05) and differentially enriched between HMR+ and HMRendo (log2 fold-change (HMRendo/HMR+) < 1.5). (TIFF) [file pgen.1009744.s003.tiff]

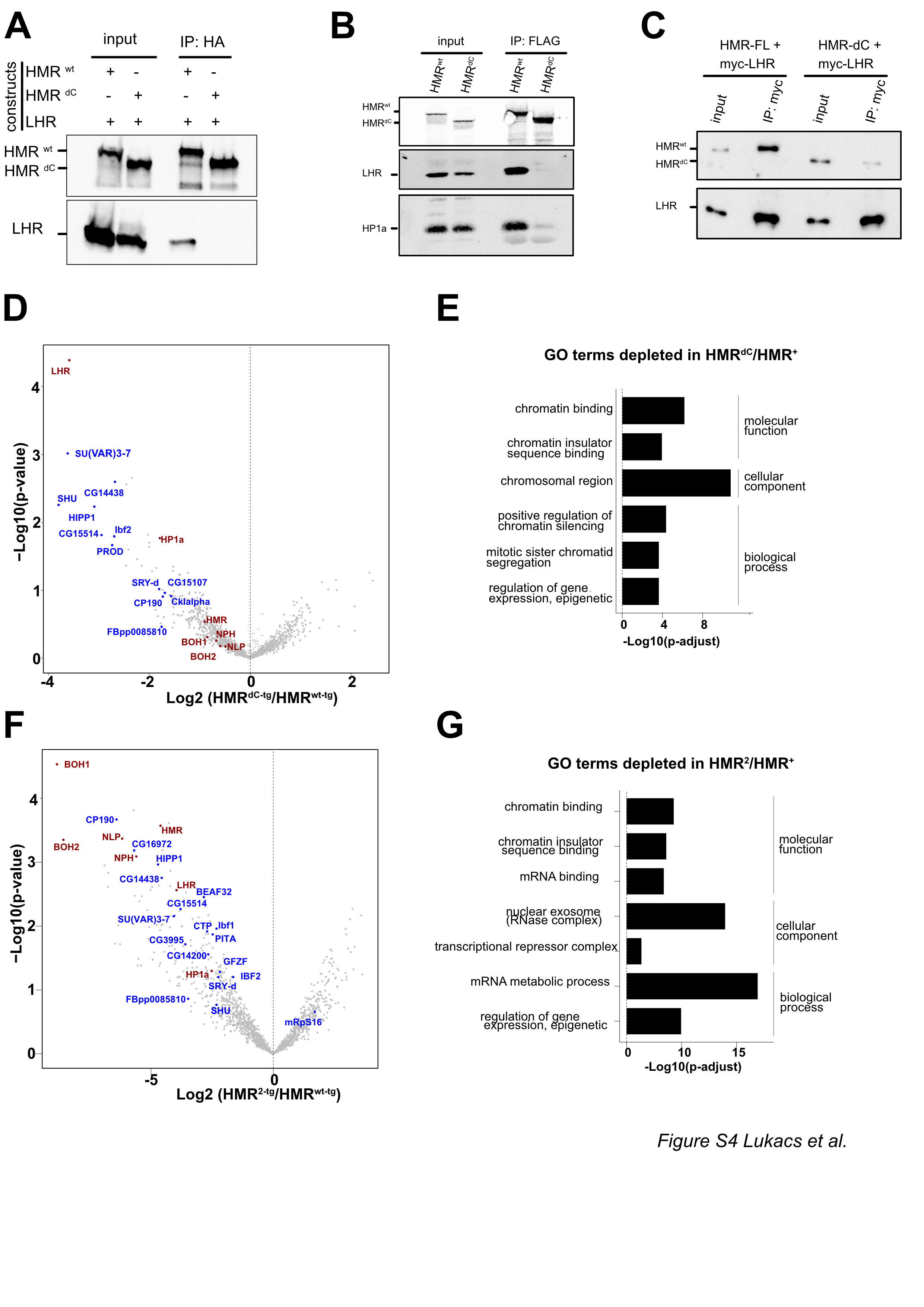

Supplement: S4 Fig — (A) Recombinantly co-expressed HMRdC and LHR do not interact. Western blot showing anti-HA immunoprecipitation in nuclear extracts from Sf21 insect cells transfected with HA-HMR and His-LHR. IP performed with anti-HA antibody and western blot probed with anti-HMR or anti-LHR antibodies. (B) HMR C-terminus is required for HMR interaction with LHR and HP1a in D. mel SL2 cells. Western blot showing HMR immunoprecipitation in SL2 cells stably transfected with either full length HMR or a C-terminally truncated HMRdC along with Myc-LHR. IP performed with anti-FLAG antibody targeting FLAG-HMR and western blot probed with anti-HA (HMR), anti-Myc (LHR) and anti-HP1a. (C) Western blot showing LHR immunoprecipitation in SL2 cells stably transfected with either full length HMR or a C-terminally truncated HMRdC along with Myc-LHR. IP performed with anti-Myc antibody targeting Myc-LHR and western blot probed with anti-FLAG (HMR) and anti-LHR. (D) Volcano plot highlighting interactions depleted in HMRdC. X-axis: log2 fold-change of FLAG-HMRdC IPs (right side of the plot) vs FLAG-HMR+ IPs (left side of the plot). Y-axis: significance of enrichment given as–log10 p-value calculated with a linear model. HMR complex subunits are labelled in red. In blue are factors depleted upon HMRdC mutation (among the endogenous or overexpression-induced interactions of HMR). Unlabeled additional bait-specific interactors are listed in S3 Table. (E) GO terms depleted upon HMRdC mutation. (F) Volcano plot highlighting interactions depleted in HMR2. X-axis: log2 fold-change of FLAG- HMR2 IPs (right side of the plot) vs FLAG- HMR+ IPs (left side of the plot). Y-axis: significance of enrichment given as–log10 p-value calculated with a linear model. HMR complex subunits are labelled in red. In blue are factors depleted upon HMR2 mutation (among the endogenous or overexpression-induced interactions of HMR). Unlabeled additional bait-specific interactors are listed in S3 Table. (G) GO terms depleted upon [file pgen.1009744.s004.tiff]

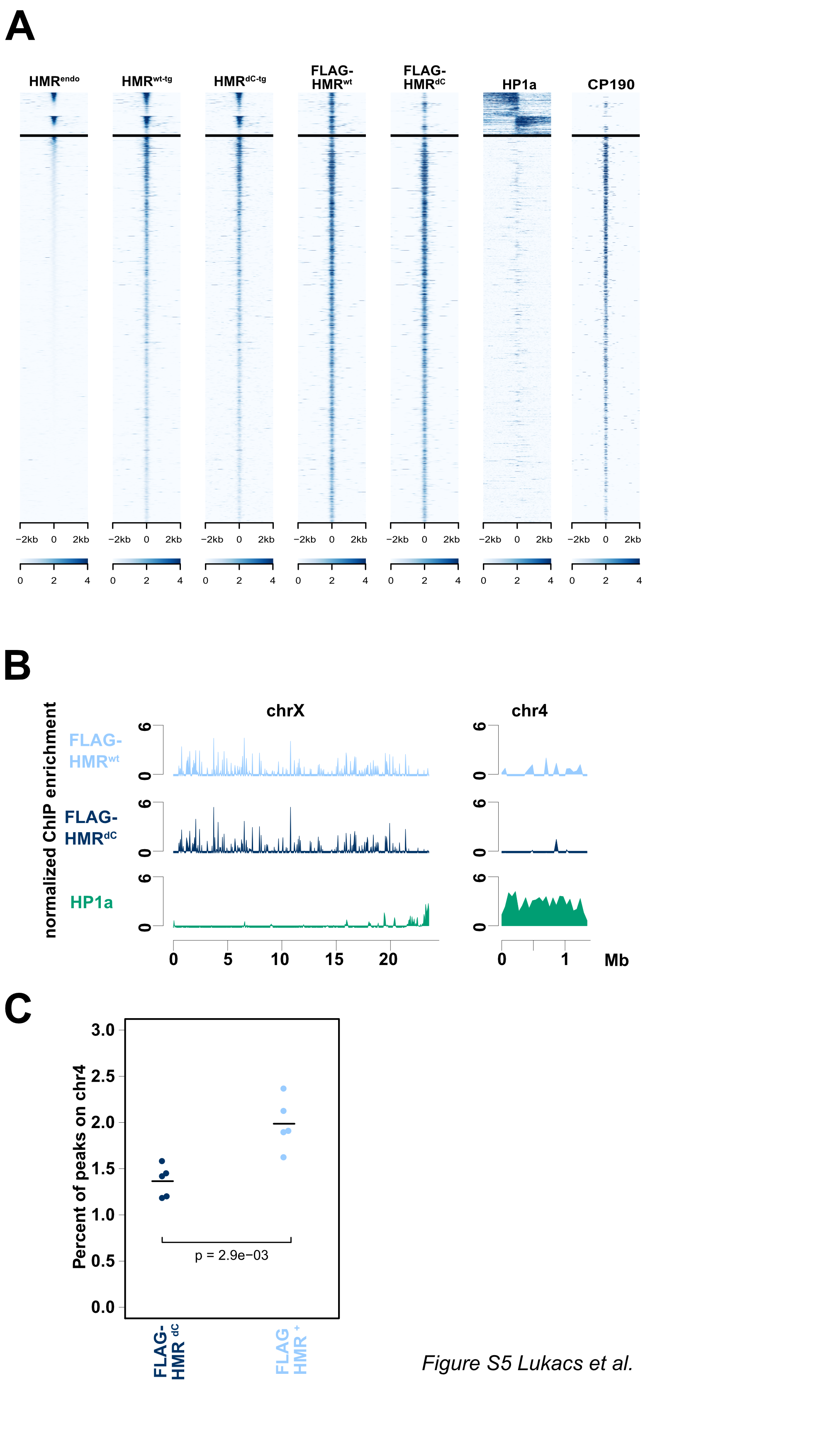

Supplement: S5 Fig — (A) Heatmaps of ChIP-seq profiles (z-score normalized) centred at high confidence FLAG-HMR peaks in 4 kb windows. Peaks are grouped by HP1a class and sorted by the ChIP signal in native HMR ChIP. From left to right, anti-HMR ChIP in untransfected cells, anti-HMR ChIP in cells transfected with FLAG-Hmr+ and FLAG-HmrdC, anti-FLAG ChIP of cells transfected with FLAG-Hmr+ or FLAG-HmrdC, and anti-HP1a and anti-CP190. The latter two are representative of the two classes of HMR peaks: HP1a-proximal and non-HP1a-proximal. (B) Chromosome-wide FLAG-HMR ChIP-seq profiles (z-score normalized) for Hmr+ (light blue), HmrdC (dark blue) and HP1a (green). Chromosomes X and 4 are shown. (C) HmrdC is depleted at heterochromatin rich chromosome 4. Percentage of FLAG-HMR ChIP-seq peaks located on chromosome 4 for each replicate (n = 5). Hmr+ (light blue) and HmrdC (dark blue) are shown. P-values are obtained by a linear model. FLAG-HMR plots represent an average of 5 biological replicates. (TIFF) [file pgen.1009744.s005.tiff]

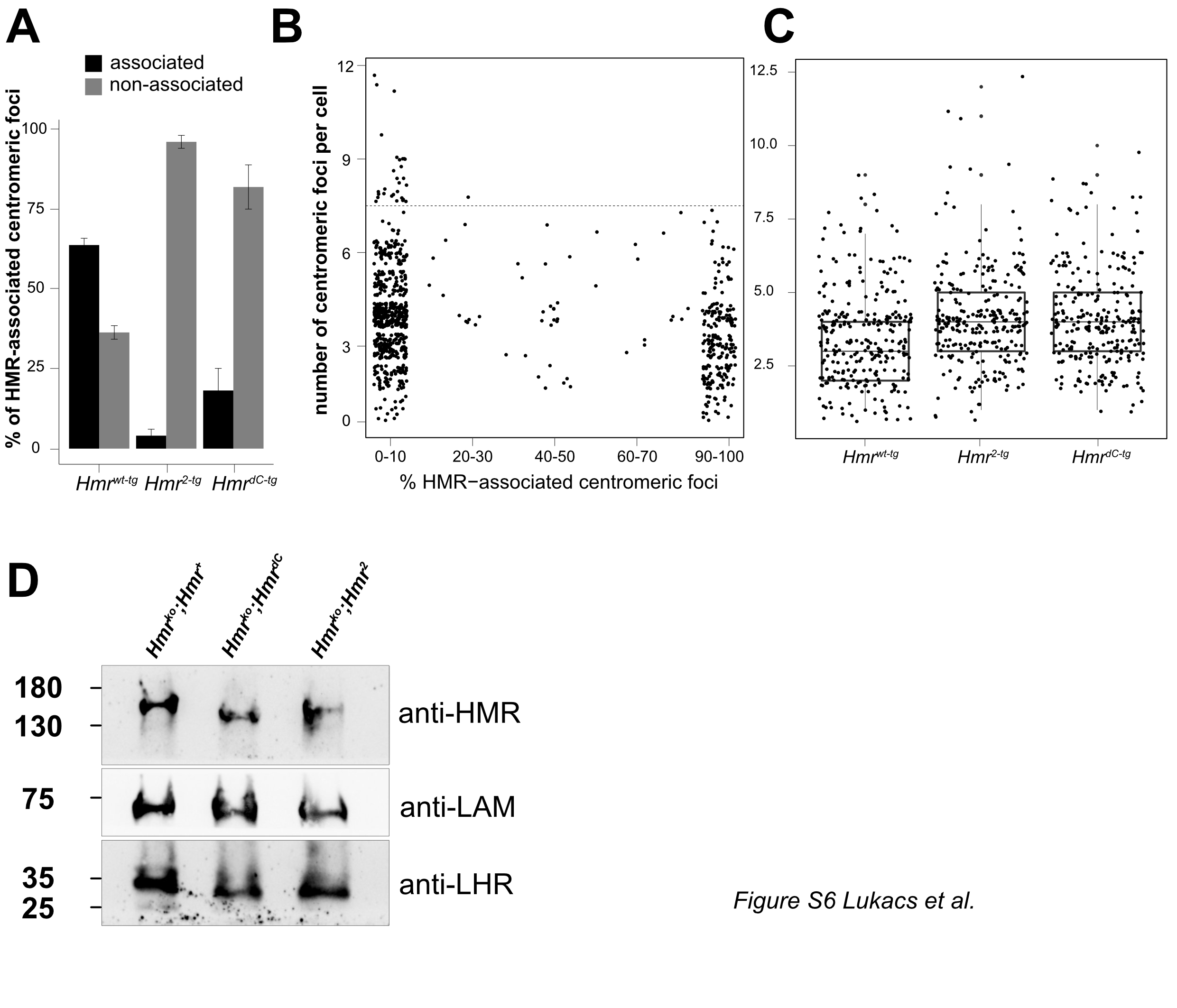

Supplement: S6 Fig — (A) The HMR C-terminus is required for HMR to form bright centromeric foci. Quantification of the percentage of centromeric foci (marked by CENP-C) associated with HMR in imunofluorescent stainings in SL2 cells expressing different Hmr transgenes (Hmr +, Hmr dC and Hmr 2). Stainings were performed with DAPI, anti-HA (recognizing HA-HMR) and anti-CENP-C antibodies. For staining details refer to Fig 4A. (B) The number of centromeric foci per cell inversely correlates with HMR’s association with centromeres. Scatter plot displaying the relation between the percentage of centromeric foci associated with HMR (x-axis, binned by 10% units) vs number of centromeric foci per cell (y-axis). Each dot represents a measured cell (a pool of all experiments from all Hmr alleles is displayed). (C) The ectopic expression of Hmr mutants correlates with higher numbers of centromeric foci. Boxplots displaying the number of centomeric foci per cell (y-axis) for each of the Hmr alleles (x-axis). Each dot represents a measured cell (a pool of all stained cells for each allele). (D) Protein expression in ovaries from Hmrko stocks complemented with different Hmr transgenes. Western blot probed with anti-HMR, anti-LHR and anti-LAMIN antibodies. (TIFF) [file pgen.1009744.s006.tiff]

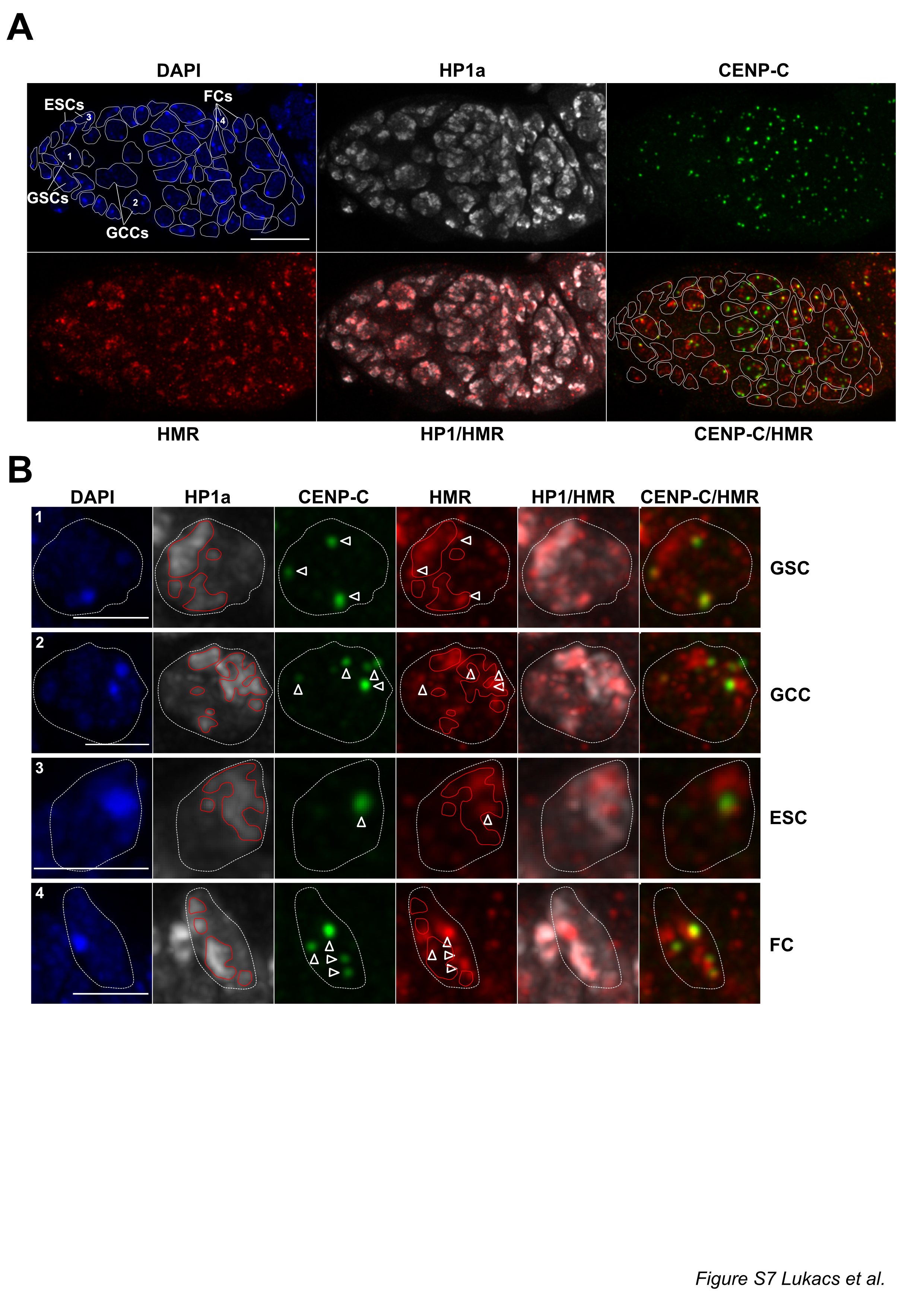

Supplement: S7 Fig — (A) Identical figure as 5A but with enlarged cells numbered. Shown are the DAPI staining (blue) and immunofluorescent stainings using anti-HP1a (white), anti-CENP-C (green), anti-HMR (red) and the merge of all channels. (B) For better illustration of the distribution of the different HMR related proteins, single cells were enlarged. In these insets the nuclear boundary is labelled with a dashed white line, the HP1a positive heterochromatic area with a red line and the centromere with a white arrowhead. Size bar indicates 10 μm in (A) and 3 μm in (B). (TIFF) [file pgen.1009744.s007.tiff]

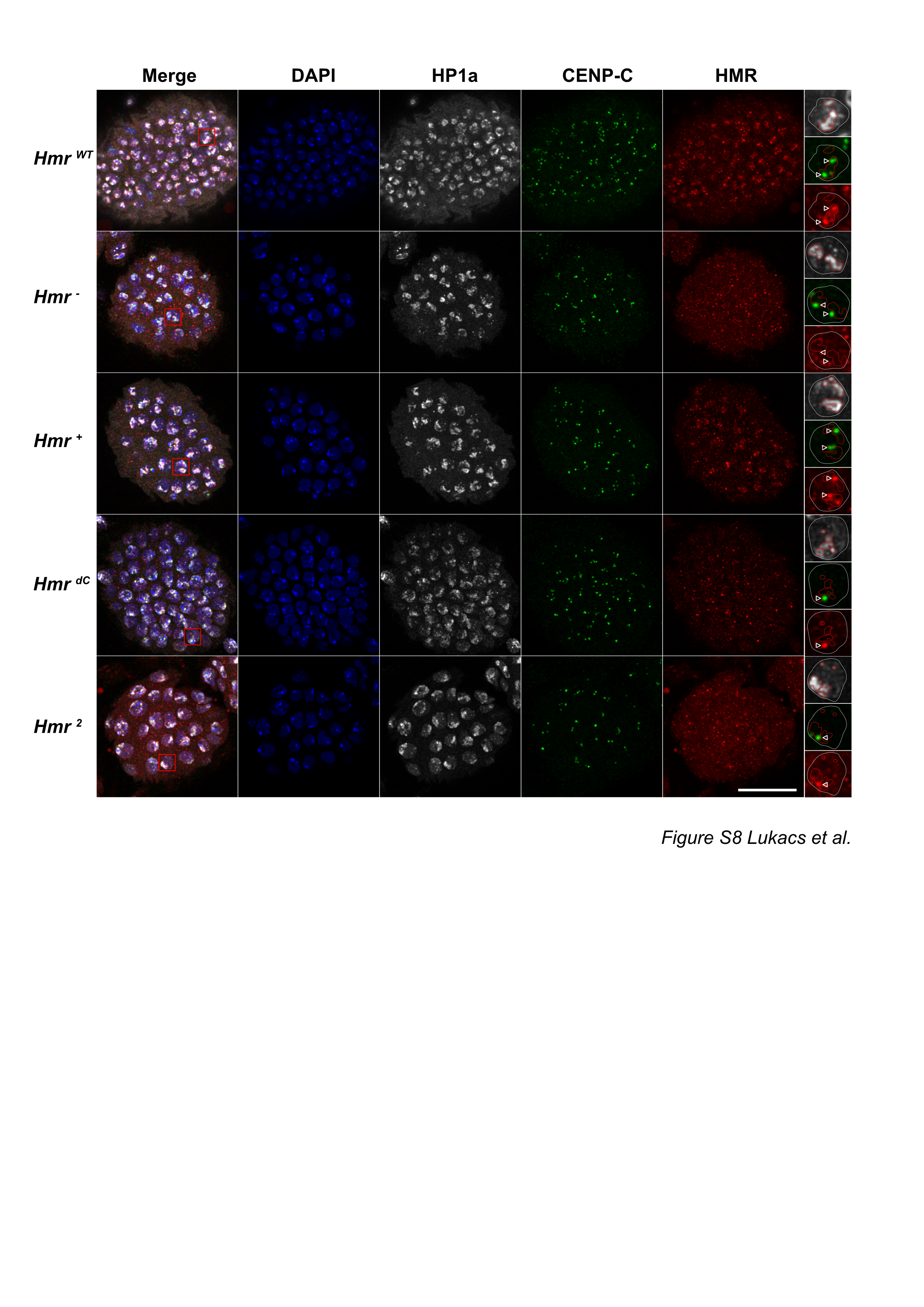

Supplement: S8 Fig — Representative images of different HMR alleles in stage 4 follicle cells. Shown are the DAPI staining (blue) and immunofluorescent stainings using anti-HP1a (white), anti-CENP-C (green), anti-HMR (red) and the merge of all channels (leftmost panels). For better illustration of the distribution of the HMR variants, a single follicle cell was enlarged and depicted on the rightmost panels. In these insets the nuclear boundary is labelled with a dashed white line, the HP1a positive heterochromatic area with a red line and the centromere with a white arrowhead. Size bar indicates 15 μm. (TIFF) [file pgen.1009744.s008.tiff]
