# Supplementary material for: The Integrity of the HMR complex is necessary for centromeric binding and reproductive isolation in Drosophila
Source: PLoS Genet. 2021 Aug 23;17(8):e1009744. doi: 10.1371/journal.pgen.1009744 (PMC8412352; doi:10.1371/journal.pgen.1009744)
Supplement: S1 Methods — (DOCX) [file pgen.1009744.s014.docx]

**SUPPLEMENTARY METHODS Lukacs et al.**

**Immunofluorescent staining in SL2 cells**

For images in S2 Fig leaky expression without copper induction was used in order to avoid overexpression artefacts. Cells were grown on #1.5H coverslips, fixed in PBS/ MeOH-free formaldehyde (4%) for 15 min at room temperature and permeabilized in PBS/Triton X-100 for 6 min on ice. After blocking with 5% normal goat serum dissolved in PBS (PBSN), coverslips were incubated with primary antibodies diluted in PBSN over night at 4°C. Fig. S2A: mouse anti-HA (Invitrogen 2-2.2.14; 1:300), rabbit anti-CID (Active Motif, 1:300), rat anti-HMR (2C10; 1:25); Fig. S2B: rat anti-HA (Sigma Aldrich 3F10; 1:100), mouse anti-HP1a (DSHB C1A9, 1:100), rabbit anti-CID (1:300). Following several washes with PBS/Triton X-100 0.05% and another blocking step with PBSN, secondary antibodies diluted in PBSN were added together with 1 µg/mL DAPI and incubated 1.5 hours at room temperature. Secondary antibodies: goat anti- rabbit Alexa Fluor Plus 647, donkey anti-rat Cy3, goat anti-mouse Alexa Fluor Plus 488. Following washes with PBS/Triton X-100 0.05% and PBS, coverslips were mounted in Prolong Diamond antifade.

**Immunofluorescent staining in ovaries**

Figs 5 and S7: All steps were performed as described in the main methods section except the following. Primary antibody solution (PBS-T, rat anti-HMR-2C10 1:20, mouse anti-HP1a-C1A9 1:10, rabbit anti-CENP-C 1:3000 and NDS 2%). Secondary antibody solution (200 µL PBS-T + donkey anti-mouse Alexa 488 1:600, donkey anti-rat Cy3 1:300, donkey anti-rabbit Alexa 647 and 2% NDS).

**Microscopy and downstream image analysis**

Fig 4A: confocal microscopy z-scans were done on a Leica TCS SP5 (with 63x objective with 1.3 NA) with a step of 0.25 µM. Sum intensities projections were analyzed, and only cells with a minimum nucleoplasmic intensity of 70 a.u. on the anti-HMR channel were taken into account for analysis. Two different quantifications were performed. In one case cells were separated and counted based on the degree of co-localization between HMR and CENP-C: overlapping, partially overlapping or non-overlapping. In parallel, the number of CENP-C marked centromeric foci associated with HMR signal was measured. Both cells and centromeric foci were blind-counted, the experiment was repeated in 2 biological replicates and for each replicate at least two slides were measured (for each slide between 24 and 63 cells were quantified).

Confocal microscopy of data presented in Figures 5C and S2 was performed at the core facility bioimaging of the Biomedical Center using the following instruments and settings:

S2 Fig: upright Leica SP8X WLL microscope (Klonike-upgraded) equipped with 405 nm laser, WLL2 laser (470 - 670 nm) and acusto-optical beam splitter. Images were acquired with a 63x/1.4 NA objective, pixel size set to 43 nm. The following spectral settings were used: DAPI (excitation 405 nm; emission 415 - 470 nm; detector: PMT), Alexa Fluor 488 (498 nm; 508 – 535 nm; HyD), Cy3 (551 nm; 561 – 610 nm; HyD), Alexa Fluor 647 (650 nm; 660 – 680 nm; HyD). Recording of image stacks was done at 200 Hz and line sequentially to avoid bleed-through. Hybrid detectors (HyDs) were operated in photon counting mode.

Fig 5C: inverted Leica SP8X STED 3D microscope (Klondike-upgraded), equipped with a 405 nm Laser, Argon Laser and a pulsed white light Laser (470 - 670 nm). Images were acquired with a 93x/1.3 NA Glycerol immersion objective, pixel size set to 63 nm. The following spectral settings were used: DAPI (405 nm; 415 - 470 nm; HyD), Alexa Fluor 488 (498 nm; 508 – 535 nm; HyD), Cy3 (551 nm; 560 – 604 nm; HyD), Alexa Fluor 647 (650 nm; 660 – 680 nm; HyD). Recording of image stacks was done at 200 Hz and line sequentially to avoid bleed-through. Hybrid detectors (HyDs) were operated in photon counting mode.

Image raw data was deconvolved using Huygens 17.10 p2. Further image processing, like generating maximum projections and linear adjustments of brightness and contrast, was done with ImageJ.
