# Supplementary material for: The Integrity of the HMR complex is necessary for centromeric binding and reproductive isolation in Drosophila
Source: PLoS Genet. 2021 Aug 23;17(8):e1009744. doi: 10.1371/journal.pgen.1009744 (PMC8412352; doi:10.1371/journal.pgen.1009744)
Supplement: Attachment — Submitted filename: 114277_1_rebuttal_2052385_qs0hyh (1).docx [file pgen.1009744.s015.docx]

Point-by point answer to reviewers:

**Reviewer 1:**

I think the experiments within the manuscript are generally of good quality and well controlled.

*We would like to thank the reviewer for the appreciation of our work.*

...However, I find that the authors' conclusions are very often not supported by the experiments performed (as detailed below) and I would strongly recommend that the authors stick to the conclusions that can be drawn based on the data they have generated. In my opinion, this manuscript contains findings that are of interest to the field but it needs to be rewritten with more justifiable conclusions.

*We have extensively rewritten the manuscript and toned down the role of the HMR/LHR complex in hybrids while emphasizing its role in Drosophila melanogaster.*

1) 'Speciation Core Complex' - The only link to speciation is the fact that the 'SCC' includes D.melanogaster HMR, a known hybrid incompatibility gene. On the other hand, all of these proteins have important functions in a pure species context and all of the interactions reported between the members of the SCC occur in a D.melanogaster background. Also, SCC assembly in viable/inviable hybrids is not tested. Essentially, I would come up with a different and more functionally consistent name for the complex. I highly recommend against naming these stable interactors as the 'SCC' unless the authors can show that mutating any of the other 'SCC' proteins (specifically NLP, NPH, BOH1 & BOH2), which should presumably also disrupt SCC formation, leads to the rescue of hybrid male lethality?

*We agree with the reviewer that we base the naming of the complex on the presence of the products of the two known hybrid incompatibility genes Hmr and Lhr. As we did not investigate the complex’ composition in hybrids we agree with the three reviewers that the term SCC is probably misleading. We also agree with the reviewer that it would be highly interesting to investigate whether NLP, NPH, BOH1 or BOH2 mutations also rescue hybrid male lethality. However, we would need to generate fly lines carrying mutations in both the D.mel and the D.sim alleles since the respective genes are autosomal and we feel that this would be beyond the scope of the manuscript. Moreover, such assays would only be possible it those genes are non-essential and not like Nlp, of which the available hypomorphic or deletion alleles are homozygous lethal (Padeken, J. et al. (2013)).*

2) Is it a stable 6-membered complex? - The only line of evidence for the presence of a stable complex between all 6 proteins are the MS data from Figure 1C and Figure S1A-C. Although I don't think it is necessarily required, a biochemical demonstration that these proteins co-sediment at a high MW would be a much stronger indication of complex formation. That being said, I think the authors can use their expertise in AP-LC/MS to more comprehensively characterize complex formation.

*Besides the fact that we observe all six components in AP-MS experiment using either one of the subunits, we have also shown in our previous experiments (Thomae et al, 2013) that all subunits can be purified by a tandem purification using first an antibody against FLAG-HMR followed by a Myc-LHR antibody. We also tried to purify the HMR complex via size exclusion chromatography to determine the size of the complex as suggested by the reviewer. Unfortunately, we did not manage to isolate enough of the complex in a soluble form that allowed us to detect a single peak on a size exclusion column. This may be either due to a disassembly of the complex during the unavoidable dilution during SEC or a lack of antibody sensitivity. We also tried to reconstitute the entire complex from recombinantly expressed proteins but failed to express all subunits in a soluble form. It is worth mentioning that a similar observation has been made, for example, for the Dosage Compensation Complex, which, despite being well characterized, has also eluded a characterization using size exclusion chromatography.*

a) For example, the authors could test whether loss of BOH1/BOH2 in S2 cells impacts complex formation. A reduction of interactions between other complex members would strengthen the authors' conclusion of a stable and stoichiometric 6-membered complex.

*Based on our observation that HMR and LHR form a stable heterodimeric complex in vitro (Figure S4) we assume that the presence or absence of the other components does not affect the complex composition in its entirety. The experiment suggested by the reviewer would allow us to distinguish between direct and indirect interactions between BOH1/2 and HMR. Though this is clearly a very exciting approach, RNAi mediated knock downs are rarely complete in S2 cells, making such experiments difficult to interpret. Therefore, these experiments would need to be supported by reconstitution of the different complexes in vitro and potentially crosslinking MS experiments. Such extensive molecular analysis would very likely require at least 6 month to be completed and would be beyond the scope of the current manuscript.*

b) Additionally, I would suggest that they use one (or more) of BOH1/BOH2/NLP/LHR as baits in the S2 cells expressing HMR mutations (HMR2 and HMR ΔC, Figure 3) to test complex formation. Beyond Figs. 1 and S1, the authors only test one-way interactions between HMR (or HMR mutants) and the other 5 binding partners. It is unclear if the other 5 'SCC' members are capable of binding each other when HMR is mutated. As a result, how HMR affects the ability of other proteins to interact with each other and its role in complex formation remains somewhat unclear. This is particularly important since the authors conclude in the discussion that "HMR acts as a molecular bridge between different modules of the SCC" and that "the integrity of the SCC is essential for its function".

*Similar to our answer to the reviewer’s suggestion above, we believe that this experiment requires an additional extensive molecular analysis to be meaningful, which is beyond the scope of the current manuscript. It is important to clarify here that the S2 cells we use still express endogenous full length-HMR, which could participate in complex formation even when Hmr mutant alleles are expressed. To unambiguously show that BOH1 and BOH2 still interact with the other complex components when they no longer associate with HMR, we would therefore need to generate a CRISPR based exchange of all HMR genes in SL2 cells with a mutated version of HMR and analyze their interaction partners. As both alleles fail to fully rescue HMR functionality in a deletion background and as we have shown previously that a removal of HMR results in mitotic defects, it may not even be possible to generate such cell lines.*

3) Centromeric vs heterochromatic localization of HMR - There appears to be some differences between Hmr localization across different tissues as the authors have noted in their introduction. In this manuscript, the authors assess HMR localization in S2 cells as well as mitotic and endocycling follicle cells from various stages of oogenesis. In these cell types, the authors compare HMR localization to both Cenp-C (centromere) and HP1 (constitutive heterochromatin). In my opinion, it is not easy to get a clear perspective on what the authors consider to be HMR's true localization in these cells and tissues. I would recommend the following straightforward changes/experiments related to this point, 

a) Label the image categories in Figure 4A. Please also describe in detail the classification criteria were used to separate these image categories from one another.

*In the revised manuscript we will label the image categories in Figure 4A. An extensive description on how the classification criteria were applied can be found in the methods section.*

b) I would also move Figure S7A to the main text since it demonstrates centromeric colocalization of HMR in early follicle cells.

*In the revised manuscript we will move figure S7A to a new figure 5C. We have furthermore investigated the localization of endogenous HMR in various cell types in ovaries, which is going to be included in the revised manuscript as a new figure 5A.*

c) Use linescans on existing images to better demonstrate colocalization between Hmr and Cenp-C and/or HP1

*In the revised manuscript we will prepare linescans/profile plots for all IF pictures when necessary.*

d) Show Cenp-A and HMR staining for the images in Figure 5C and stage 10 follicle cells from Figure S7A.

*As stainings with the Cenp-C antibody resulted in more stable and reproducible signals, we used Cenp-C as a proxy for Cenp-A and centromere localization. In Figure S7A and B we stained Cenp-C and showed a greatly reduced expression in follicle cells undergoing endoreplication. We therefore did not perform a Cenp-C (or Cenp-A)/HMR co-staining in these cells and do not think it would add to a better understanding of the mechanisms of HMR locaization (Figure 5C).*

e) I feel the authors do not spend enough time discussing the fact that HMRΔC still appears to localize to centromeres at most follicle cells upto Stage 7.

*We now also include the staining of endogenous HMR (figure 5A revised ms) in the various cell types in ovaries. This allows us to expand the discussion of HMR’s localization in dependency of the cell type and stage. These studies not only reveal the high diversity of HMR localization but also suggests that the potential of HMR to localize to the centromere as well as pericentromeric heterochromatin is crucial for its function. In the revised manuscript we have now discussed the fact that HMRdC still localizes to the centromere up to stage 7 more extensively*.

In sum, it would also be nice for the authors to take a clear position on whether HMR is centromeric, heterochromatic or both in the cells they analyze by microscopy and why these localizations may change between the cells they have looked at.

*The fact that we now include a novel figure where we investigate HMR’s localization in different cell types allows us to discuss the (diverse) localization as well as its potential regulation more extensively. As the localization is highly dependent on the cell type observed as well as the cell cycle stage use, we feel that these aspects need to be taken into account when describing HMRs localization. This is now discussed in the revised manuscript.*

4) HMR2 analyses - I think HMR2 is an important mutant to include as a control for HMRΔC, especially since the authors should already have the required strains/data. I specifically mean the following,

a) Figure 4C - Please add HMR2 ChIP-seq tracks only if the authors already have this data.

*Unfortunately, we were unable to acquire convincing HMR2-ChIP data. This may be due to the fact that HMR^2^ localizes quite diffusely or due to a lower percentage of cells expressing this allele in the S2 lines used. Both issues do not influence our interpretations in AP-MS experiments or in single cell based fluorescence microscopy assays, but is problematic in bulk cell population assays like ChIP. Therefore, we cannot provide good HMR^2^ ChIP-Seq tracks.*

b) Figure 5C and Figure S7B - Add HMR2 IF images. Please also discuss HMR2 localization to centromeres and heterochromatin.

*In the revised manuscript, we have/will attache(d) IF images of ovarial tissue made from strains heterozygous for the Hmr^2^ allele. Due to the lower gene dosage the intensity of HMR stainings is reduced making a precise localization more difficult. As the manuscript mainly focusses on the description of the newly discovered HmrdC allele, we have added this as supplemental material.*

c) Figure 5E - Increase n's for the HMR2 fertility assay.

*The HMR^2^ allele has been extensively characterized by Aruna and colleagues (Aruna et al., Genetics  (2009)) with regards to its effect on fertility. For this particular assay we only use it as a positive control and reference for the newly described HMRdC allele. We therefore feel that an increase in the number of replicates would be redundant to the earlier publications*.

5) HMR localization in female germline cells - Given that the authors indicate that female fertility and telomeric transposon suppression are compromised with HMR2 and HMRΔC, I think it would strengthen the manuscript to address HMR localization with respect to heterochromatin and centromeres in the nurse cells and/or oocytes.

*We now also include the staining of endogenous HMR (figure 5A revised ms) in nurse cells, oocytes and early-stage follicle cells. This allows us to expand the discussion of HMR’s localization in dependency of the cell type and stage.*

6) I find the last part of the abstract and discussion i.e. HMR bridges heterochromatin and the centromere, to be very speculative based on the data presented. As far as I can tell, the only experimental basis for this conclusion is the fact that HMR binds known centromeric and heterochromatic proteins. With this logic, you could easily make a similar argument for the numerous proteins that colocalize with centromeric and pericentromeric heterochromatin. Personally, I would not speculate extensively on a HMR bridging activity without more compelling functional readouts.

*Our hypothesis of HMR as a bridging factor between centromeric and pericentromeric heterochromatin is not only based on its colocalization and interaction with components of chromatin types but also on our previous findings that an HMR knockdown results in a moderate centromere declustering and studies using super-resolution microscopy, which indicate that HMR is sandwiched between the two components (Kochanova, N. Y. et al. (2020)). As the proteomic analysis of the two HMR alleles presented in this study suggest that interactions with both components are required for full functionality of HMR, we assume that it bridges between the two chromatin components. However, we agree with the reviewer that this could also be explained by a centromeric as well as a heterochromatic function of HMR, which are independent from each other. We therefore removed the hypothesis from the abstract and discussed it together with other potential explanations for our findings.*

**Minor comments:** 

1) Intersection plot - I would explain the intersection plot on Figure 1C more thoroughly (I found it confusing).

*We expanded the paragraph in which we explain the intersection plot in figure 1C.*

2) Image colours - The images in Figure S2 and Figure S7 are hard to interpret due to the colours used for the HA and Hmr channel respectively. I would use the white pseudo-colour for DAPI and omit this channel from the merged image and insets (a line demarcating the nucleus would suffice in the merged image). In addition, a linescan would better represent colocalizations or lack thereof.

*We have omitted the DAPI channel from the merged images and used a line to demarcate the nucleus as suggested by the reviewer. To better illustrate co-localisation of distinct factors we will used line profile plots.*

3) I'm not convinced that one can determine stoichiometry and sub-stoichiometry of protein complexes based on spectral counts; spectral counts could be affected by other factors. Therefore, I would hesitate to use "However, HP1a is only present in sub-stoichiometric amounts in the AP-MS purifications with antibodies against the SCC...."

*The question of whether the stoichiometry of complexes using iBAQ values of purified protein complexes is intensely discussed in the field. Several studies do suggest that this can indeed be done (i.e. Wohlgemuth, Iet al. Proteomics* ***15****, 862–879 (2015); Smits, A. H., Nucleic Acids Research* ***41****, e28–e28 (2012)), which is why we commented on the lower intensity of HP1a relative to the other subunits of the complex. However, we agree with the reviewer that this can only be an approximation rather than a precise measurement (which would need a full in vitro reconstitution, see comments above). We have mentioned this in the revised manuscript.*

4) Ambiguity in description of methods - In the methods section 'Crosses for generating Hmr genotypes for hybrid viability assays', the authors state that "In the rescue experiment, Hmr+ served as a positive (lethality rescue) and Hmr2 as a negative control (no lethality rescue)". The authors might consider rewording this as I think it's a bit strange to refer to hybrid male lethality as a rescued state.

*We agree with the reviewer that the wording to describe the assay we used to investigate HMR’s function in male hybrids is counterintuitive as a “rescue of functionality” results in male hybrid lethality. To better describe it we now call the assay “hybrid viability suppression”, according to the nomenclature that has been used by Aruna et al, 2009 (Aruna, S. et al. Genetics (2009)).*

.

Reviewer #1 (Significance (Required)): 

**Nature and Significance of the advance:** 

This work adds to the study of reproductive isolation in Drosophila by defining a stable set of molecular interactors of the HMR hybrid incompatibility protein. In my opinion, this study offers a platform for future research into the poorly understood molecular events that trigger hybrid incompatibility in Drosophila. In addition, the authors generate a novel HMR mutation (HMRΔC) that also rescues hybrid male lethality and it would be interesting to determine in finer detail how closely this mutation mimics other known HMR mutations. A characterization of BOH1/BOH2 would have also significantly strengthened the manuscript.

*We would like to thank the reviewer for the appreciation of our work. We agree with the reviewer that a deeper characterization of BOH1/BOH2 will further unravel their role in the complex. However, our initial experiments using null alleles or knock downs of BOH1 and BOH2 in D.mel showed no effect or only minor effects on transposon activation and hybrid male lethality. This is most probably due to the fact that the D.sim alleles can fully complement for their function. Moreover, the recombinant expression of BOH1 and 2 turned out to be difficult due to problems in protein solubility. We therefore need to postpone our BOH1 and 2 studies to a later timepoint.*

**My Expertise:** 

Satellite DNA repeats, Chromocenters, Speciation, Hybrid Incompatibility 

**Referees cross-commenting" 

I also agree that all the reviewer comments are reasonable. The manuscript would be significantly improved by making conclusions that can be supported by the data. I think some additional experiments are also warranted to make the paper more robust. 


Reviewer #2 (Evidence, reproducibility and clarity (Required)): 

In this study, the authors identify a protein complex that contains hybrid incompatibility genes Hmr and Lhr, naming it SCC (Speciation Core Complex). 
This paper's major conclusions are: 1) overexpression of Hmr (which resembles the situation in hybrid, where hmr/lhr are overexpressed) results in ectopic protein-protein interaction. 2) Hmr's DNA binding domain (mutated in Hmr2) and C-terminal domain (known to interact with Lhr) are important for its function and in causing hybrid lethality. 

The identification of SCC complex is quite intriguing, but this paper does not cover much of functional significance of this complex at all. For example, does mutating other components of SCC complex (BOH1 etc) rescue hybrid lethality? Without examining these important issues, they instead drifted to study the domain function of Hmr. It is not so clear why these two lines of studies are glued together in one paper. 

It is not that I insist that the authors have to do all these experiments, but the assembly of the paper makes this paper quite inconclusive. After reading it, the readers are left behind wondering what is the function of SCC---and we do not even know whether 'speciation core complex' is a fair naming, without any knowledge whether any of the components being involved in speciation or not. 

Overall, this work contains a lot of important information, which promises future breakthrough on the subject matter. However, unfortunately, the study is not carried out to generate any conclusion and is fairly incomplete at this point.

*We thank the reviewer for his appreciation of the importance of our work and apologize that we did not clarify the reasoning of the experiments sufficiently. We think that part of the reviewer’s disappointment is due the fact that we named the complex speciation core complex (SCC), which was indeed an unfortunate decision as we are unable to investigate the complex in male hybrids where it exerts it’s function in mediating hybrid incompatibility (see also answer to comments of reviewer 1). We therefore changed the name to HMR complex and tried to better explain the rational of our experiments in the text.*

**Specific comments.** 

- Quality of Fig4A is too low. I cannot even tell where is the boundary of nucleus. Diffuse signal in category 'yellow' and 'grey'---are they entire cell or nucleus or nucleolus? Please add additional marker(s) for better interpretation of the Hmr signal presented.

*We have improved the quality of figure 4A by adding lines to indicate the nuclear boundary and inserting profile plots to better illustrate the different types of co-localisation.*

- In Fig4A and 5C, the localization of Hmr (wild type version) looks quite different in these two images. Which image is more 'representative' for Hmr localization? (as they build the logic on Hmr localization, this inconsistency is quite bothering). This might be cell-type-specific issue, but if so, how do we know the relevance of their localization? These issues make the result of localization analysis of wt/mutant Hmr inconclusive.

*After reading the reviewers responses we realized that we did not describe our findings well enough, which resulted in a major confusion about the localization of HMR in cells. Indeed, the localization of HMR differs widely depending on the cell type used. We have now included a new figure (new Figure 5A) illustrating the analysis of the endogenous HMR localization in ovaries isolated from D.mel. We hope that the additional figure together with our interpretation helps to alleviate the confusion and adds to the understanding of HMR’s function and potential evolution of HMR.*

Reviewer #2 (Significance (Required)): 

Hmr and Lhr are known as 'hybrid incompatibility genes', deletion of which rescues male hybrid lethality in Drosophila melanogaster/simulans hybrid crosses. Understanding the molecular function of Hmr and Lhr is expected to provide insights into the fundamental question of how two species become incompatible (i.e. how speciation occurs). This study investigates the protein complex that contains Lhr and Hmr, identifying a previously unidentified 'core' complex. Understanding the function of this complex may significantly advance our understanding of speciation. 

**Referees cross-commenting" 

I think all review comments are reasonable. 
However, I'd like to emphasize that the biggest issue with this paper is not about the data, but how the authors frame it. The term such as 'speciation core complex' is beyond 'hype' (not even 'exaggeration'). Simply there is no evidence that this term can be supported. I think the authors need to be more ethical. I would be surprised if authors truly believe they can claim that the term 'speciation core complex' is justifiable in science. 


Reviewer #3 (Evidence, reproducibility and clarity (Required)): 

**Summary:** 

The manuscript "The integrity of the speciation core complex is necessary for centromeric binding and reproductive isolation in Drosophila" by Lukacs and colleagues describes a study that show, by mass-spec and ChIP-seq, that two well established hybrid incompatibility proteins form a 6-protein complex that predominantly localizes near HP1a bound chromatin boundaries. With a C-terminal domain of HMR deleted, the 6-protein core complex was not disrupted, but its interaction and subsequent localization to HP1a domain near centromeres was lost. In addition, an HMR double mutant that disrupts the interaction between HMR and other components of the 6-protein core complex was tested and similar distribution patterns as for the dC mutant were observed. Next, the nuclear localization was HMR was tested in fruit fly follicle cells by IF. In endoreplicating cells, HMR-dC did not colocalize with HP1a, as did the double mutant. The expression level of several transposable elements (TEs) was assessed and only the full length wt Hmr transgene was able to rescue the repression of TEs, whereas neither the dC and double mutants did. When the number of offspring was assayed, a similar pattern was observed. Finally, male hybrid lethality was assayed by crossing D melanogaster mothers with different Hmr alleles with wt D simulans and only the wt Hmr allele resulted in male lethality, whereas both cD and double mutants resulted in 10-40% of the offspring to be male. These findings led the authors to conclude that 1) 6-protein speciation core complex containing HMR, LHR, NLP, NPH, and two uncharacterized proteins called BOH1 and BOH2, 2) overexpression of HMR/LHR results in novel interactions with other chromatin factors, 3) both the double mutant (E317K and G527A) and the C-terminal deletion mutant are important for for protein-protein interaction within the 6-protein complex and associated factors such as HP1a, and 4) HMR bridges heterochromatin and centromeres. 

**Major comments:** 

+ Most of the key conclusions are supported by the evidence presented in this manuscript. The link between centromeres and HMR (and presumably the rest of the 6-protein complex) hinges only on colocalization IF and ChIP-seq data. The change in Hmr localization in cycling follicle vs endoreplicating cells of especially the dC mutant is very interesting. The loss of CENP-C signal correlates with a change in Hmr^dC signal. What exactly drives this change is not explored.

*We have shown in the past that HMR requires full length Cenp-C to localize to the centromere in S2 cells. We assume that this is also the case in the follicle cells. Therefore, the lack of Cenp-C recruitment in endoreplicating cells is likely the reason why HMR localizes primarily to HP1a containing heterochromatin. Differently from wild type HMR, HMRdC can’t bind LHR/HP1a as our AP-MS data show and therefore is not recruited to heterochromatin and diffuses away in later stages. We have described this point more extensively in the revised manuscript*

+ The data presented in this manuscript are mostly clear (see minor comments) and appear to be reproducible, especially as the methods sections is detailed and both the ChIP-seq and mass-spec data is deposited in publicly accessible databases. 
+ The rational why both HMR and LHR are overexpressed in cell lines is not clearly explained.

*As outlined in our response to reviewer 1 the overexpression of HMR and LHR was designed to simulate the hybrid situation, which shows an increase in HMR and LHR levels (Thomae, A. W. et al.  Developmental Cell* ***27****, 412–424 (2013)). We have indicated this in the revised manuscript.*

+ The HMR/LHR overexpression experiment is very nice, and as one would expect, resulted in more protein interactions. Some of these might simply be the result from the abundance of HMR and LHR, which have saturated the core 6-protein complex. This leaves the question what is the true minimal size of the HMR/LHR complex? The dC mutant that removes the BESS domain as well as the double point mutations that disrupts the complex altogether, get to the importance of the stability of the complex and its association with especially HP1a. What the minimal interacting partners of HMR and LHR could be explored by knocking-down both factors and do mass-spec.

*We agree with the reviewer that the abundance of HMR and LHR results in a saturation of the core complex thereby having a spillover effect on other proteins. In this regard it is worth mentioning that the expression of the Hmr^2^ allele does not completely disrupt the complex but rather results in a loss of interactions with NLP, NPH, BOH1 and BOH2 while maintaining the interaction with LHR and HP1a. In fact, when the HMR^2^ protein is expressed, it shows a stronger interaction with known heterochromatic proteins than the wt protein (Figure 3B). As both mutant alleles show functional defects in pure species and in male hybrids we assume that HMR and LHR need to bind both chromatin types simultaneously. We consider the complex to be somewhat modular as we show that HMR and LHR can interact in isolation (Figure S4) while others have shown that LHR and HP1a, as well as NLP and NPH interact (Greil, F. et al. EMBO J (2007); Anselm, E. et al. Nucleic Acids Research (2018) respectively). This is now pointed out in the revised manuscript*

+ For the telomeric TE expression as well as offspring count shown in Figure 5D,E, a wild-type control would be informative as a measure how well the Hmr+/+ rescues both phenotypes.

*The misregulation of transposable elements (TE) and fertility defects of Hmr loss of function mutants have been previously characterized (Satyaki, P. R. V. et al.  PLoS genetics (2014); Aruna et al.,Genetics  (2009)). We therefore rather focused on the relative expression of TEs in the HmrdC and Hmr2 mutants relative to the wild type rescue allele (Hmr+). Hmr2 serves as a known non-rescue allele (Aruna et al., 2009) in the fertility experiment, while in the TE experiment we describe for the first time a defect in TE repression for this allele.*

**Minor comments:** 

+ In the opening paragraph of the introduction, the authors describe a scenario of sympatric speciation, which is subsequently highlighted by the speciation event between D. melanogaster and D. simulans. Yet, these two species have similar but not identical distribution range, leaving open the possibility the speciation event happened in parapatry. It might be worth rephrasing the first paragraph to leave open both modes of speciation, especially as the manuscript focuses on the mechanistic side of hybrid incompatability-associated proteins.

*We did not want to imply that our experiments allow a distinction between a sympatric or parapatric speciation. We thank the reviewer for pointing this out and rephrased the first paragraph accordingly.*

+ Some of the abbreviations are repeated (e.g. SCC) others aren't introduced (e.g. HI). Overall, less abbreviations will make the text more readable, especially for non-experts.

*We tried to avoid acronyms wherever possible and got rid of the term SCC altogether. All acronyms are introduced at the first appearance.*

+ In IF signal in Figure 4A is difficult to see on the black background. I would suggest either increasing the gain to improve the visibility of the signal or show in black-and-white. In addition, the colors should be labeled in the figure for clarity.

*We improved the quality of Figure 4A and labeled the different types of localization (see also answer to reviewer 1).*

+ In Figure 5C the images for the Hmr^KO;Hmr^2 appears to be missing.

*See answer to reviewer 1 (4b). We have/will include the corresponding picture as supplementary material as we consider the characterization of the novel Hmr allele to be the main focus of the manuscript.*

In addition, for non-experts it might be helpful to mention which set of IF images are controls, rescues, and test, similar to what was done in Figure 5B.

*We have/will indicate which IF pictures are controls and rescue experiments*

Reviewer #3 (Significance (Required)): 

**Significance:** 

+ This study provides novel insight how two factors involved in male hybrid lethality, with which chromatin factors they are associated, and how two mutants impact the chromatin localization and in vivo phenotypes. 
+ Understanding the molecular basis of speciation is limited as most factors that drive speciation are not identified. Drosophila species are at the forefront of this research. Post-zygotic factors have predominantly found to have strong speciation potential. This work build very nicely on this work. 
+ This manuscript will be predominantly interesting for the Drosophila chromatin field and speciation field. 
+ I am trained in comparative genomic focusing on centromeric repeats and now study chromatin dynamics at the single molecule level, using cell biology, biochemical and biophysical tools.

*We thank the reviewer for appreciating our work. We think that our work will also be interesting for researchers focusing on centromere clustering and genome organization in general and independently of the Drosophila system.*

**Referees cross-commenting" 

Reviewer comments look reasonable to me- 1-3 months revision is not an undue burden, I think they can do at least some of what was requested. 
In response to Rev2: Agreed, they ought to tone it down 
