# Supplementary material for: The Integrity of the HMR complex is necessary for centromeric binding and reproductive isolation in Drosophila
Source: PLoS Genet. 2021 Aug 23;17(8):e1009744. doi: 10.1371/journal.pgen.1009744 (PMC8412352; doi:10.1371/journal.pgen.1009744)
Supplement: Attachment — Submitted filename: Review_PLoS Genetics.docx [file pgen.1009744.s017.docx]

The authors have improved their manuscript and I don’t have any objections to its publication. I have noted down some minor comments to improve data presentation as well as some typos/grammatical errors that I caught.

Minor comments

1) Figure 5 could be further improved as follows,
   a. Label the individual channels in 5A (as in figure 6)
   b. Label the stages and/or cell types for 5B-D
   c. Insets for GSC/ESC/GCC/FC in 5A would make co-localization data more apparent
2) Response to Reviewer #1, comment 4c – ‘Figure 5E - Increase n's for the HMR2 fertility assay’.

I take the authors point that *Hmr^2^* has been previously well-characterized but n=2 for a fertility assay doesn’t do any favours for the rest of the figure. I would omit this data since *Hmr^KO^* is already present as a positive control.

Typos/Grammatical Errors

1) Abstract – ‘~~a~~ decreased fertility’. This line could also use some clarification since it sounds like the authors are describing decreased fertility of hybrids (which are sterile).
2) Author summary – ‘die because the**y** contain elevated levels of this complex…’
3) Author summary – ‘mutations that interfere with the ~~complexes~~ complex’s function...’
4) Introduction (page 3 second paragraph, last line) – ‘multiple novel binding~~s~~ sites**,** many of which have been previously…
5) Figure S2 – Inconsistency between usage of ‘Cid’ in the figure legend and Cenp-A in the figure.
6) Results (page 16, first paragraph, penultimate line) – ‘To have a more comprehensive *<missing word>* of HMR’s…..’
7) Discussion (page 19, second paragraph, penultimate line) – ‘both our ChIP Seq data and ovaries stainings in early follicle cells…’ I would reword ‘early’ to ‘S7’ to be more specific.
