# Supplementary material for: The Integrity of the HMR complex is necessary for centromeric binding and reproductive isolation in Drosophila
Source: PLoS Genet. 2021 Aug 23;17(8):e1009744. doi: 10.1371/journal.pgen.1009744 (PMC8412352; doi:10.1371/journal.pgen.1009744)
Supplement: Attachment — Submitted filename: Point-by point response.docx [file pgen.1009744.s018.docx]

Point-by point answer to the reviewers

**Reviewer #1:** 

Overall, these data imply that both spatiotemporal regulation of chromatin factors and interaction with other chromatin-associated factors are critical for establishing and maintaining chromatin function and subsequent downstream effects. It will be interesting to learn how future studies will tease apart how the binding of HMR to these two chromatin domains are regulated. The updated discussion succinctly captures this point.

We thank the reviewer for her appreciation of our work

On page 16, the sentence “To have a more comprehensive of HMR’s localization in flies …" does not flow well

We corrected this to: “To have a more comprehensive view”

On page 17, the use of the word “flies” in “… can also be observed in flies, we wanted …” is a little broad. Maybe write out more specifically which fly they are referring to?

The intention of this sentence was to contrast the results done in living flies to tissue culture cells. To better clarify we replaced “flies” with “the developing ovaries of *D. melanogaster”*

Reviewer #2:

The authors have improved their manuscript and I don’t have any objections to its publication. I have noted down some minor comments to improve data presentation as well as some typos/grammatical errors that I caught.

Minor comments

Figure 5 could be further improved as follows,

a. Label the individual channels in 5A (as in figure 6)
b. Label the stages and/or cell types for 5B-D
c. Insets for GSC/ESC/GCC/FC in 5A would make co-localization data more apparent

We realized that insets for the different cell types in 5A would either make the figure too large or the insets too small. We therefore added a new supplementary figure S7, in which we show enlarged images of the different cell types and labeled the cell types in 5B-D.

I take the authors point that *Hmr^2^* has been previously well-characterized but n=2 for a fertility assay doesn’t do any favours for the rest of the figure. I would omit this data since *Hmr^KO^* is already present as a positive control.

Following the reviewer’s suggestion, we removed the data of Hmr2 fertility assay

Typos/Grammatical Errors

Abstract – ‘~~a~~ decreased fertility’. This line could also use some clarification since it sounds like the authors are describing decreased fertility of hybrids (which are sterile).

We change this sentence to “The overexpression of the two proteins also leads to mitotic defects, a misregulation in the expression of transposable elements and decreased fertility in pure species.”

Author summary – ‘die because the**y** contain elevated levels of this complex…’

Author summary – ‘mutations that interfere with the ~~complexes~~ complex’s function...’

Introduction (page 3 second paragraph, last line) – ‘multiple novel binding~~s~~ sites**,** many of which have been previously…

Figure S2 – Inconsistency between usage of ‘Cid’ in the figure legend and Cenp-A in the figure.

We corrected all the typos and grammatical errors and apologize for the oversight

Results (page 16, first paragraph, penultimate line) – ‘To have a more comprehensive *<missing word>* of HMR’s…..’

We corrected this to: “*To have a more comprehensive* ***view***”

Discussion (page 19, second paragraph, penultimate line) – ‘both our ChIP Seq data and ovaries stainings in early follicle cells…’ I would reword ‘early’ to ‘S7’ to be more specific.

We corrected this to: “*Additionally, ovary stainings in follicle cells up to stage S7,…*”

**Reviewer #3:** 
In previous version(s?) of this manuscript, the reviewers generally agreed that the data quality is good but the interpretations are not well-supported. In this revised version, the authors have improved the writing considerably, and the overall conclusions are reasonably supported. Specific comments below cover several additional issues along this line, but once they are addressed, this manuscript will be of considerable interest for the field.

Page 13-14: They infer that the pulldown experiment under Hmr overexpression condition mimics hybrid condition. However, it should be noted that this is still in pure species condition (absence of any simulans proteins). Of course, they don’t need to do such experiments, but this difference needs to be clearly mentioned.

We now point out explicitly that the overexpression experiments are done in SL2 cells made from Drosophila melanogaster (in pure species conditions).

Figure2, referring Hmr ectopic expression to as ‘Hmr+’ is very misleading. I understand this means ‘wild type transgene’, but in the context of differentiating Hmr-endo vs. Hmr+, it’s hard to understand ‘Hmr+’ indicates ectopic expression.

We agree with the reviewer that the labelling of ectopically expressed HMR as HMR+ can be misleading. We therefore labeled all ectopically expressed transgenes as HMR^*-tg^ (wt-tg, 2-tg,dC-tg), while maintaining the labeling of HMR expressed from a different locus in flies as HMR^+^ for the wt and HMR^dC^ for the dC mutant allele. We hope that the reviewer agrees with us that this is the most comprehensive way of labeling.

Please label Figure 5A.

We labeled the figure 5A and added a new supplementary figure S7 with insets of individual cells from the germarium.

Fig7 title ‘Hmr C-ter is required for hybrid lethality’ is misleading. Although this statement is not wrong, the results might simply indicate that Hmr-deltaC is just non-functional, as is shown in Figure 6. But the figure title/subheading indicate a little more than that (as if it is specifically required for hybrid lethality, without affecting its endogenous function in pure species context). I recommend a slightly more cautious description.

We change the title of the figure to “Deletion of *Hmr’s* C-terminus results in failure to rescue hybrid lethality”
